# The monkey microbial biobank brings previously uncultivated bioresources for nonhuman primate and human gut microbiomes

**DOI:** 10.1002/mlf2.12017

**Published:** 2022-05-24

**Authors:** Danhua Li, Chang Liu, Rexiding Abuduaini, Mengxuan Du, Yujing Wang, Haizhen Zhu, Honghe Chen, Nan Zhou, Yuhua Xin, Linhuan Wu, Juncai Ma, Yuguang Zhou, Yong Lu, Chengying Jiang, Qiang Sun, Shuang‐Jiang Liu

**Affiliations:** ^1^ State Key Laboratory of Microbial Resources and Environmental Microbiology Research Center at Institute of Microbiology Chinese Academy of Sciences Beijing China; ^2^ College of Life Science University of Chinese Academy of Sciences Beijing China; ^3^ State Key Laboratory of Microbial Biotechnology Shandong University Qingdao China; ^4^ China General Microorganism Culture Collection, Institute of Microbiology, Chinese Academy of Sciences Beijing China; ^5^ Microbial Resources and Big Data Center, Institute of Microbiology, Chinese Academy of Sciences Beijing China; ^6^ State Key Laboratory of Neuroscience, CAS Key Laboratory of Primate Neurobiology CAS Center for Excellence in Brain Science and Intelligence Technology, Chinese Academy of Sciences, Institute of Neuroscience Shanghai China

## Abstract

Nonhuman primates (NHPs) such as monkeys are the closest living relatives to humans and are the best available models for causative studies of human health and diseases. Gut microbiomes are intensively involved in host health. In this study, by large‐scale cultivation of microbes from fecal samples of monkeys, we obtained previously uncultured bacterial species and constructed a *Macaca fascicularis* Gut Microbial Biobank (MfGMB). The MfGMB consisted of 250 strains that represent 97 species of 63 genera, 25 families, and 4 phyla. The information of the 250 strains and the genomes of 97 cultured species are publicly accessible. The MfGMB represented nearly 50% of core gut microbial compositions at the genus level and covered over 80% of the KO‐based known gut microbiome functions of *M. fascicularis*. Data mining showed that the bacterial species in the MfGMB were prevalent not only in NHPs gut microbiomes but also in human gut microbiomes. This study will help the understanding and future investigations on how gut microbiomes interact with their mammalian hosts.

Gut microbiomes (GMs) contribute to human health and diseases^1^. Nonhuman primates (NHPs) are the evolutionary‐closest living relatives and share high similarities in genetics, anatomy, physiology, and behavioristics to human beings^2^. Thus, the NHPs such as *Macaca fascicularis* are ideal models for the study of human diseases with intricate pathogenesis and phenotypes that would be hard to be replicated in other mammalian animals. For instance, many studies concerning the development and treatment of mental disease, for example, autism disease and Parkinson's disease, were performed using NHPs, and the experimental results were more reliable to be extrapolated to humans^3^, ^4^. Although the taxonomic composition and strain colonization of gut microbiota usually exert host preference^5^, ^6^, ^7^, previous results revealed that captivity humanizes the NHPs gut microbiota^8^, ^9^. In recent years, species of the genera *Bifidobacterium* ^10^, *Helicobacter* ^11^, *Aeromonas* ^12^, *Lactobacillus* ^13^, *Limosilactobacillus gorillae* ^14^, and *Alloscardovia theropitheci* ^15^ were isolated from NHPs. Although the microbial ecology of the gastrointestinal tract of the rhesus monkey (*Macaca mulatta*) has been studied early in 1971,^16^ less efforts were made on the cultivation and collection of gut microbial resources from NHPs^12^, ^13^, ^15^ compared with human^5^, ^6^, ^17^, ^18^, ^19^, mouse^7^, ^20^, and pig^21^. According to the analysis of six available NHPs metagenomic cohorts, many gut microbial species had not been cultivated and were unexplored due to the shortage of reference genomes and the lack of cultured bioresources^22^. Thus, an extensive collection of cultivable gut microbes from NHPs is of practical importance and would (1) facilitate causative studies of host–microbe interactions, (2) develop new interventions for GMs dysbiosis, and (3) promote the in‐depth comparison of human and NHPs GMs.

In this study, we constructed a *Macaca fascicularis *Gut Microbial Biobank (MfGMB; homepage: https://nmdc.cn/mfgmb/ and http://www.cgmcc.net/english/mfgmb/) that consisted of 250 strains representing 97 different species of 63 genera, 25 families, and 4 phyla. MfGMB harbored 32 novel species that were characterized and denominated by following the rules of the International Code of Nomenclature of Prokaryotes. Based on taxonomic studies, 13 novel genera and 1 novel family were proposed to accommodate the new bacterial species. In silico analysis revealed that the newly characterized bacterial taxa were prevalent in both monkey and human guts, and the MfGMB genomes covered over 80.0% of the known function (KEGG Orthologs) of *M. fascicularis* gut global gene catalog^22^.

The construction of MfGMB was initiated by large‐scale cultivation of gut microbes, and totally, 73 culture conditions (Materials and Methods, and Datasheets S1 and S2 in Supporting Information) were employed to cultivate microbes from 16 fecal samples. More than 7000 colonies were collected for enlarged cultivation and 16S ribosomal RNA (rRNA) genes were sequenced. As a result, 4100 pure bacterial isolates were obtained. The isolate IDs, their closest phylogenetic relatives, and 16S rRNA gene sequences are provided (Supporting Information Datasheet S3). According to 16S rRNA gene sequence identity (a cutoff value of 98.7%), 4100 isolates were further phylogenetically clustered into 97 different taxa (Supporting Information Datasheet S5). One strain was selected to represent each taxon for further studies. Thus, 97 representative strains were obtained and their genomes were sequenced (Supporting Information Datasheet S6). The quality and purity of 97 representative genomes were evaluated using CheckM (Supporting Information Materials). The 97 genomes were of good quality, as the average completeness of assemblies reached 97.06 ± 6.84% (median value was 99.18%), the average contamination was 0.96 ± 1.47% (median value was 0.43%), and the mean value of the estimated quality score (completeness −5 × contamination) was 92.25 ± 9.63% (median value was 95.98%). Of the 97 strains, we found that 32 strains did not phylogenetically match any previously known species and represented potentially novel bacterial taxa. We then characterized the 32 strains in terms of their cell morphology, DNA sequence‐based phylogeny and phylogenomy, genomic analysis, and BIOLOG tests as described in Methods (see Supporting Information Materials). With this polyphasic taxonomy, results showed that all 32 strains were recognized as novel species, of which 18 belonged to previously described genera, 13 represented new genera, and 1 represented a new family. All new taxa were denominated following the rules of the International Code of Nomenclature of Prokaryotes (ICNP)^23^ and their protologues are provided in Table 1, and eight of the novel species have been described in detail^24^. Finally, the MfGMB comprising 250 strains of 97 species from 63 genera, 25 families, and 4 phyla were deposited at the China General Microbiological Culture Collection Center (CGMCC) for public accessibility. The taxonomic diversity of MfGMB is displayed in Figure 1A and the detailed information of all 250 representative strains (e.g., original strain IDs/names, genome features, accession numbers, etc.) are provided in Supporting Information Datasheet S4 and also with MfGMB homepage (https://nmdc.cn/mfgmb/and http://www.cgmcc.net/english/mfgmb/). The 97 representative genomes are publicly accessible via NCBI and NMDC (see Data Availability).

**Table 1:** The protologues of 32 novel species in MfGMB.

Taxonomy	Type designation	Description^a^	GMCC accession
*Zhongyuia ovalis*	MSJ‐20^T^ from monkey feces	Cells are spherical or oval, nonmotile. The genomic DNA G + C content of the type strain is 30.65 mol%.	CGMCC 1.31770
*Zhichengia intestinisimiae*	MSJ‐13^T^ from monkey feces	Cells are rod‐shaped, nonmotile. The genomic DNA G + C content of the type strain is 36.15 mol%.	CGMCC 1.32902
*Baoxiangia intestinisimiae*	MSJ‐15^T^ from monkey feces	Cells are rod‐shaped, nonmotile. The genomic DNA G + C content of the type strain is 33.91 mol%.	CGMCC 1.32904
*Xuanrenia ovalis*	MSJ‐17^T^ from monkey feces	Cells are oval to rod‐shaped, nonmotile. The genomic DNA G + C content of the type strain is 49.63 mol%.	CGMCC 1.32906
*Hualania flagellata*	MSJ‐21^T^ from monkey feces	Cells are oval to spherical, motile. The genomic DNA G + C content of the type strain is 43.69 mol%.	CGMCC 1.32910
*Tongshengia biacuta*	MSJ‐24^T^ from monkey feces	Cells are spindle‐shaped, nonmotile. The genomic DNA G + C content of the type strain is 39.81 mol%.	CGMCC 1.32912
*Taichongia intestinisimiae*	MSJd‐27^T^ from monkey feces	Cells are rod‐shaped, nonmotile. The genomic DNA G + C content of the type strain is 44.49 mol%.	CGMCC 1.45014
*Zhenyingia intestinisimiae*	MSJ‐30^T^ from monkey feces	Cells are rod‐shaped, nonmotile. The genomic DNA G + C content of the type strain is 55.4 mol%.	CGMCC 1.32917
*Huanchunia intestinalis*	MSJ‐31^T^ from monkey feces	Cells are rod‐shaped, nonmotile. The genomic DNA G + C content of the type strain is 58.46 mol%.	CGMCC 1.32918
*Shaojiongia intestinisimiae*	MSJ‐32^T^ from monkey feces	Cells are rod‐shaped, nonmotile. The genomic DNA G + C content of the type strain is 29.02 mol%.	CGMCC 1.32919
*Qingshengia brevis*	MSJ‐33^T^ from monkey feces	Cells are rod‐shaped, nonmotile. The genomic DNA G + C content of the type strain is 42.74 mol%.	CGMCC 1.32920
*Huaguia intestinisimiae*	MSJ‐37^T^ from monkey feces	Cells are rod‐shaped, nonmotile. The genomic DNA G + C content of the type strain is 62.17 mol%.	CGMCC 1.32924
*Youchengia simiae*	MSJ‐38^T^ from monkey feces	Cells are long rod‐shaped, motile. The genomic DNA G + C content of the type strain is 55.01 mol%.	CGMCC 1.32925
*Yanxiongia mobilis*	MSJ‐39^T^ from monkey feces	Cells are curved spindle‐shaped, motile. The genomic DNA G + C content of the type strain is 43.93 mol%.	CGMCC 1.32926
*Peptoniphilus ovalis* ^24^	MSJ‐1^T^ from monkey feces	Cells are spherical, nonmotile. The genomic DNA G + C content of the type strain is 30.65 mol%.	CGMCC 1.31770
*Dysosmobacter acutus* ^24^	MSJ‐2^T^ from monkey feces	Cells are rod‐shaped, nonmotile. The genomic DNA G + C content of the type strain is 58.27 mol%.	CGMCC 1.32896
*Amphibacillus intestinalis*	MSJ‐3^T^ from monkey feces	Cells are rod‐shaped, nonmotile. Growth in modified GAM medium occurs at 37°C, pH: 7.0–7.5, in 3–5 days. The genomic DNA G + C content of the type strain is 36.18 mol%.	CGMCC 1.32897
*Clostridium simiarum* ^24^	MSJ‐4^T^ from monkey feces	Cells are rod‐shaped, motile. The genomic DNA G + C content of the type strain is 30.46 mol%.	CGMCC 1.45006
*Alkaliphilus flagellates* ^24^	MSJ‐5^T^ from monkey feces	Cells are rod‐shaped, motile. The genomic DNA G + C content of the type strain is 31.71 mol%.	CGMCC 1.45007
*Paenibacillus brevis* ^24^	MSJ‐6^T^ from monkey feces	Cells are rod‐shaped, motile. The genomic DNA G + C content of the type strain is 49.3 mol%.	CGMCC 1.45008
*Butyricicoccus intestinisimiae* ^24^	MSJd‐7^T^ from monkey feces	Cells are spherical, nonmotile. The genomic DNA G + C content of the type strain is 50.29 mol%.	CGMCC 1.45013
*Clostridium puyanii*	MSJ‐8^T^ from monkey feces	Cells are rod‐shaped, nonmotile. The genomic DNA G + C content of the type strain is 28.63 mol%.	CGMCC 1.32898
*Blautia simiae*	MSJ‐9^T^ from monkey feces	Cells are rod‐shaped, nonmotile. The genomic DNA G + C content of the type strain is 41.07 mol%.	CGMCC 1.32899
*Clostridium mobile* ^24^	MSJ‐11^T^ from monkey feces	Cells are rod‐shaped, motile. The genomic DNA G + C content of the type strain is 30.38 mol%.	CGMCC 1.45009
*Roseburia mobilis*	MSJ‐14^T^ from monkey feces	Cells are rod‐shaped, motile. The genomic DNA G + C content of the type strain is 38.62 mol%.	CGMCC 1.32903
*Blautia beijingensis*	MSJ‐19^T^ from monkey feces	Cells are rod‐shaped, nonmotile. The genomic DNA G + C content of the type strain is 42.89 mol%.	CGMCC 1.32908
*Anaerostipes simiae*	MSJ‐23^T^ from monkey feces	Cells are oval‐shaped, nonmotile. The genomic DNA G + C content of the type strain is 35.74 mol%.	CGMCC 1.32911
*Ornithinibacillus simiae*	MSJ‐26^T^ from monkey feces	Cells are rod‐shaped, motile. The genomic DNA G + C content of the type strain is 36.67 mol%.	CGMCC 1.32914
*Dysosmobacter brevis*	MSJ‐29^T^ from monkey feces	Cells are short rods, nonmotile. The genomic DNA G + C content of the type strain is 57.84 mol%.	CGMCC 1.32916
*Paenibacillus difficilis*	MSJ‐34^T^ from monkey feces	Cells are rod‐shaped, nonmotile. The genomic DNA G + C content of the type strain is 50.7 mol%.	CGMCC 1.32921
*Blautia ovalis*	MSJ‐36^T^ from monkey feces	Cells are long rod‐shaped, nonmotile. The genomic DNA G + C content of the type strain is 44.06 mol%.	CGMCC 1.32923
*Tissierella simiarum* ^24^	MSJ‐40^T^ from monkey feces	Cells are rod‐shaped, motile. The genomic DNA G + C content of the type strain is 30.39 mol%.	CGMCC 1.45012

^a^ Growth in modified GAM medium occurs at 37°C, pH: 7.0–7.5, in 3–5 days. MfGMB, *Macaca fascicularis* Gut Microbial Biobank.

**Figure 1:**
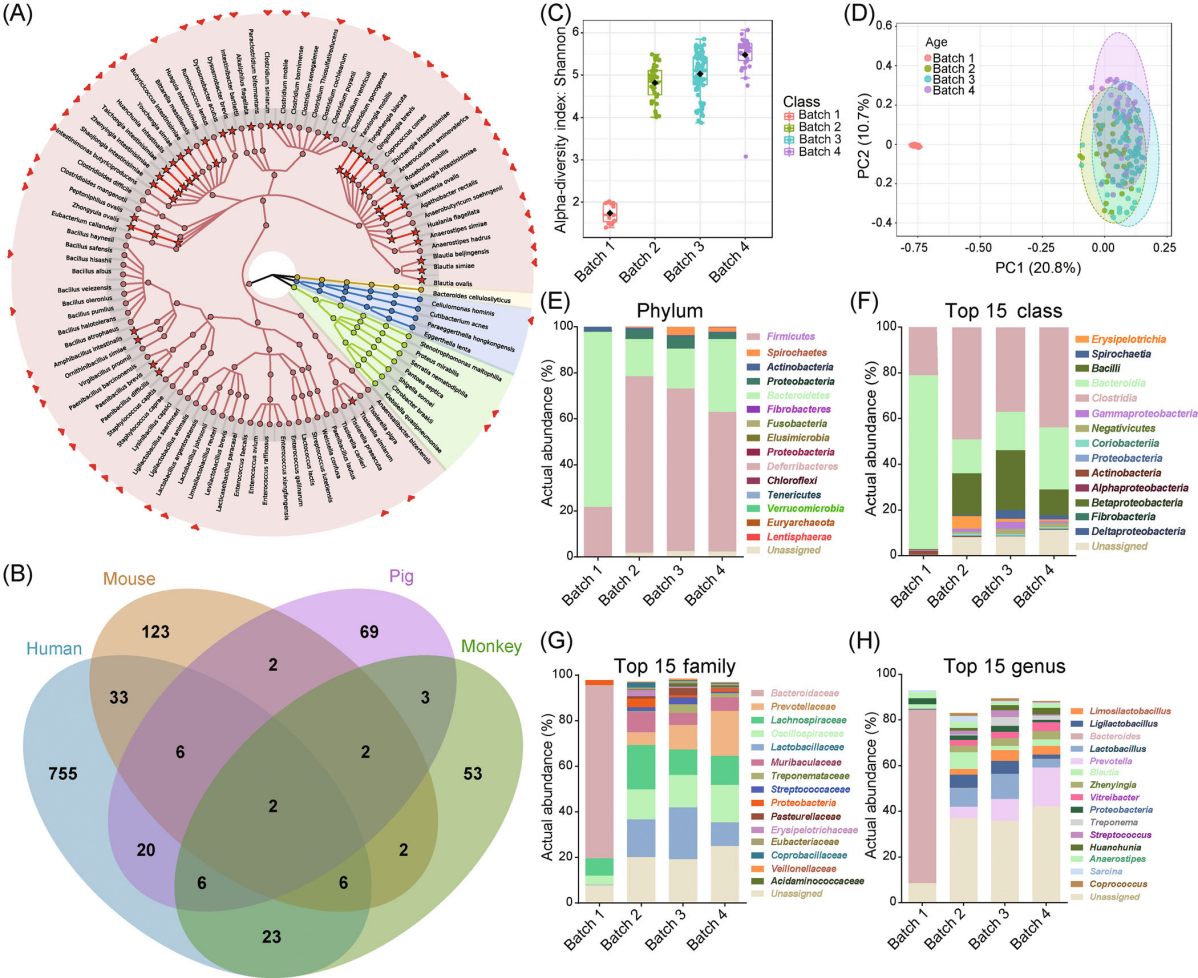
The taxonomic diversity of *Macaca fascicularis* Gut Microbial Biobank (MfGMB) and of fecal samples (*n* = 161) from *M. fascicularis*. (A) The taxonomic cladogram of MfGMB. The novel taxa at family, genera, and species levels are represented by red stars. The outer ring shows the unique 53 species that are solely covered by MfGMB and are labeled with red triangles. The background is color‐coded according to four phyla. (B) The Venn diagram displays the numbers of shared and specific species in mammalian gut microbial collections. The culture collections from human, mouse, pig, and *M. fascicularis* are, respectively, marked as Human, Mouse, Pig, and Monkey and are differently colored. (C) Shannon index illustrating the alpha‐diversity of gut microbiota in fecal samples collected in different batches of isolations. (D) Principal coordinate analysis (PCoA) plot displaying the beta‐diversity of gut microbiota in fecal samples collected in different batches of isolations. (E–H) The actual abundance of phyla (E), top 15 abundant classes (F) and families (G) and genera (H) of different batches of sampling. The 16S rRNA gene amplicon dataset used in this analysis was generated from 161 fecal samples from 7 *M. fascicularis* sampled at different time points (0–5 years); Batch 1: 13 fecal samples from two 2‐month‐old experimental *M. fascicularis*, Batch 2: 39 fecal samples from two 6‐month‐old experimental *M. fascicularis*, Batch 3: 75 fecal samples from four 10‐month‐old and two 5‐year‐old experimental *M. fascicularis*, Batch 4: 34 fecal samples from two 1‐year‐old and two 5‐year‐old experimental *M. fascicularis*. The PCoA plot was statistically analyzed with ANOSIM, the *p* < 0.001. rRNA, ribosomal RNA.

We compared the compositions of MfGMB at species‐level with gut microbial collections derived from human^5^, ^6^, ^17^, ^18^, ^19^, mouse^20^, ^25^, and pig^21^, and found that the MfGMB had unique species and expanded mammalian gut microbial biobanks. By a combined analysis of all the gut microbial culture collections from the same host, 851, 176, 110, and 97 gut microbial species were cultured from human, mouse, pig, and monkey, respectively. The distribution and overlap of species in host‐specific collections are displayed in Figure 1B, and it is noted that 44 shared species (45.36%) and 53 (54.64%) unique species out of the 97 MfGMB species were included by MfGMB (Figure 1A). The 53 unique species of monkey gut belonged to 42 genera, and these 53 unique species expanded the mammalian gut microbial collections. We noted that 13 of the 53 unique microbial species represented core genera (for a definition of “core genera,” see the following paragraphs) as detected by 16S rRNA amplicon sequencing, which was characteristic and host‐specific for monkey as dominant communities.

To assess the microbial diversity of gut microbiota of *M. fascicularis* and evaluated the representativeness of the MfGMB to the fecal microbial diversity in this study, we sequenced the 16S rRNA gene amplicons of 161 fecal samples from *M. fascicularis* collected at different time points that were used for four batches of large‐scale bacterial isolation works. The datasets were processed with a standard USEARCH‐based analysis pipeline and were annotated using LTP_vbiobank customized by supplementation of LTP database with the 16S rRNA gene sequences of 32 novel taxa as described in the Methods (see Supporting Information Materials). First, the alpha diversity of gut microbiota in Batch 1 was significantly lower than that of the other three groups (Figure 1C), while as shown in Figure 1D, the beta diversity of Batch 1 was also distant from the other three groups. Moreover, the taxonomic divergence between Batch 1 and the other three groups was also observed by the taxonomic annotation‐based analysis (Figure 1E–H). Specifically, at the phylum level (Figure 1E), 97.8 ± 1.3% of the total reads were assigned into 15 different phyla, while the relative abundance (RA) of Firmicutes and Bacteroidetes were the most dominant, and the two phyla accounted for 92.6% of the total reads. Notably, Bacteroidetes (RA = 76.0 ± 6.4%) were the most dominant phylum followed by Firmicutes (RA = 21.5 ± 6.0%) in Batch 1. Yet, in the other three batches, Firmicutes was the most abundant phylum (RA = 76.6 ± 12.0% for Batch 2, 70.6 ± 13.7% for Batch 3, and 60.6 ± 13.6% for Batch 4), and Bacteroidetes was the second dominant one with RA values ranging from 16.1% to 31.8%. When we examined the lower taxa of the datasets, a similar conclusion was drawn. The diversity of Batch 1 differed from the other three batches of samples at class, family, and genus levels (Figure 1F–H). The differences of taxonomic and compositional diversity were possibly ascribed to the change of diet from formula milk at 2‐month age (Batch 1) to normal feed after 6‐month old (Batches 2, 3, and 4), and the use of samples from hosts at different life stages might facilitate a better recovery of diverse gut microbes from experimental *M. fascicularis*.

Subsequently, to further evaluate the taxonomic representativeness of MfGMB to the gut microbiota of *M. fascicularis*, we compared the taxa of MfGMB with the combined 16S rRNA amplicon datasets (samples from four different batches were merged together, *n* = 161) at the genus level. The results revealed that 60.6 ± 14.9% of the total reads were assigned into 155 genera, and the MfGMB covered 31 of them. If we defined the genera with average frequency of occurrence (FO) > 80% as “common genera,” those genera with average RA > 0.1% as “dominant genera,” and those genera shared by both “common” and “dominant” cohorts as “core genera,” then 47, 38, and 37 genera were recognized as common, dominant, and core genera, respectively (Figure 2A). The MfGMB covered 38.3%, 47.4%, and 48.6% of the common, dominant, and core genera, respectively. There were five newly described genera (*Zhengyingia* gen. nov., *Huachunia* gen. nov., *Shaojiongia* gen. nov., *Qingshengia* gen. nov., and *Baoxiongia* gen. nov.) belonging to the core genera.

**Figure 2:**
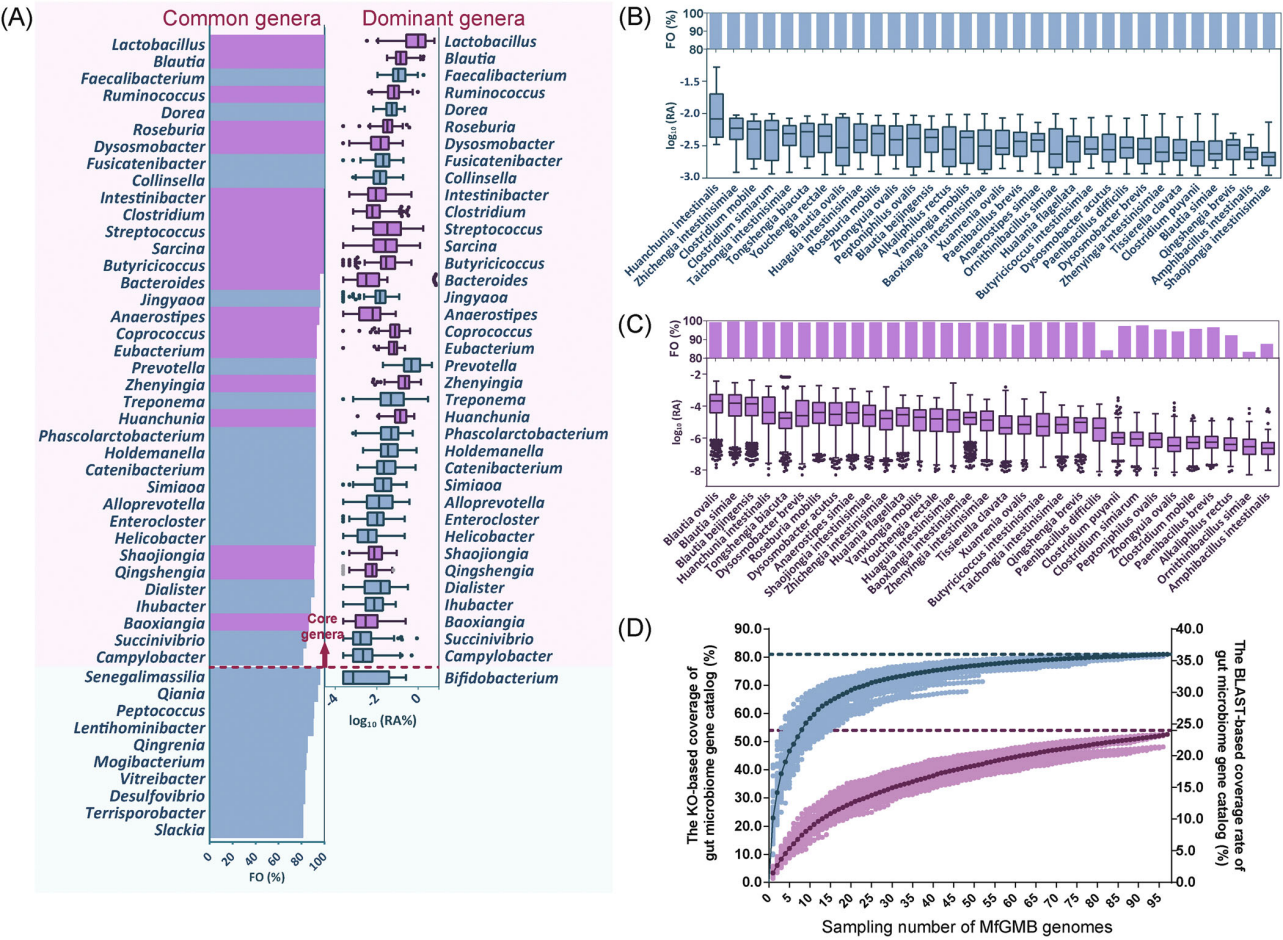
The representativeness, prevalence, distribution, and coverages of *Macaca fascicularis* Gut Microbial Biobank (MfGMB) species and genes. (A) The representativeness of major genera of *M. fascicularis* gut microbiota (*n* = 161) by MfGMB. Common genera: genera with average frequency of occurrence (FO) > 80% (definition: FO = 100% is defined when a taxon presents in all samples, while FO = 0 is defined when a taxon presents in none of the samples). Dominant genera: genera with average relative abundance (RA) > 0.1%. The light‐pink background in the panel highlights the core genera shared by both dominant and common genera, while the light‐blue background marks out the taxa presenting uniquely in either dominant or common genera. The bar chart shows the FO of each common genus (%), while the Box‐and‐Whiskers plot shows log_10_ of RA (%) of each dominant genus. The prevalence and abundance of new taxa in gut microbiomes of *M. fascicularis* (*n* = 25) (B) and humans (*n* = 1129). (C) In panels, the bar chart displays the FO of each new species (%), while the Box‐and‐Whiskers plot shows log_10_ of RA (%). (D) The rarefaction curve displaying the KEGG Ortholog (KO)‐based (blue) and BLAST‐based (purple) accumulatively increased coverage of *M. fascicularis* gut microbiome by MfGMB genomes. The sampling was repeated 100 times at each *x*‐axis point. Light blue dot: the coverage rates of KO functions of *M. fascicularis* gut microbiome global gene catalogs when specified numbers of genomes were randomly sampled from 97 MfGMB genomes. Dark blue line: the mean coverage rate of KO functions. Light purple dot: the coverage of the gene sequences of *M. fascicularis* gut microbiome global gene catalogs by the randomly sampled genomes with threshold values of 40% identity and 70% coverage. Dark purple line: the mean coverage rate of aligned sequences. Center line: median, bounds of the Box: quartile, Whiskers: Tukey extreme.

Thirty‐two of the 97 species in MfGMB represented new taxa. To demonstrate the prevalence of these new taxa in NHPs and the human GMs, first, we analyzed 25 available *M. fascicularis* gut metagenome samples as described in Methods (see Supporting Information Materials). In total, 39.52 ± 17.73% reads were annotated into 8409 species. As shown in Figure 2B, all the 32 novel taxa were found in 25 samples (FO = 100%), and the RA ranged from 0.0025 ± 0.0016% (*Shaojiongia intestinisimiae* gen. nov., sp. nov.) to 0.15 ± 0.15% (*Huanchunia intestinalis* gen. nov., sp. nov.). Second, we analyzed the distribution of the 32 new taxa in human GMs by Kraken2‐based annotation of 1129 metagenomes of healthy human fecal samples. It manifested in that the 32 new taxa widely existed also in human guts (Figure 2C). The FO for all the new taxa and the mean value of FOs reached >80% and up to 96.97%, respectively, which indicated that the newly characterized taxa were prevalent in both monkey and human guts. Noticeably, of the 32 new taxa, the top three abundant species (*Blautia ovalis* sp. nov., *Blautia simiae* sp. nov., and *Blautia beijingensis* sp. nov*.*) were all from the same genus *Blautia*, while *Huanchunia intestinalis* gen. nov., sp. nov., as the most abundant new MfGMB taxa in monkey GMs (Figure 2B), ranked the fourth richest in humans (Figure 2C). Moreover, we also compared the genomes of the 32 new taxa with over 1000 metagenome‐assembled genomes (MAGs) representing previously uncharacterized species of NHP GM constructed recently using six publicly available NHP metagenomic cohorts^26^. It revealed that 15 of our new taxa got hit on MAGs, while four of them represented previously uncultured “dark” taxa without any reference genome ever achieved before this study.

To further reveal the functional potentials of genomes in MfGMB, we created a gene catalog containing 313,603 nonredundant genes with 97 MfGMB genomes (named MfGMB.catalog) and compared it by BLAST analysis against the *M. fascicularis* global gut microbial gene catalog containing 1,991,169 nonredundant genes constructed by Li et al.^26^ (named global catalog). The results showed that the MfGMB catalog covered 463,647 of the proteins in the global catalog at 40% identity and 70% coverage. It drastically enriched the existing global catalog by 123,771 new genes, as only 189,832 genes were shared by both catalogs. We then investigated the representativeness of MfGMB genomes to the annotated functions of *M. fascicularis* GM. For this purpose, the 97 MfGMB genomes and the global gene catalog were annotated with eggNOG 5.0^27^. Of the 97 genomes, 180,019 genes were annotated into 6803 different KEGG Orthologs (KOs), while 955,272 genes of the global catalog were annotated into 7075 different KOs. A cumulative analysis of the KO profiles was conducted to determine the coverages of the global catalog by a random incremental selection of the 97 genomes. As shown in rarefaction curves, the MfGMB genomes covered 5733 of the KO genes from global catalogs (blue lines in Figure 2D) accounting for 81.0% of the known function of *M. fascicularis* GM represented by the global gene catalog. If we quantify the representativeness of MfGMB genomes to the GM at the sequence level rather than the functional level, the 97 MfGMB genomes represented 23.3% of the gene sequences at 40% amino acid identity and 70% sequence coverage. Besides the good recovery of functionally known genes, each MfGMB genome harbored an average of 40.11 ± 9.28% genes of unknown functions (Supporting Information Datasheets S7 and S8), indicating the potential roles of MfGMB as a cultivable gene pool for the culture‐dependent study of “dark” functions in *M. fascicularis* GMs.

In summary, we constructed a microbial biobank, the MfGMB, for monkey GMs. The information on 250 bacterial strains of 97 species as well as their genome data is publicly available at the MfGMB homepage (https://nmdc.cn/mfgmb/and http://www.cgmcc.net/english/mfgmb/). The MfGMB covered nearly 50% core genera of GM samples (*n* = 161) from monkeys aged 0–5 years. In addition to the characteristic bacterial taxa that are represented by the 13 core genera, including five novel genera (*Zhengyingia* gen. nov., *Huachunia* gen. nov., *Shaojiongia* gen. nov., *Qingshengia* gen. nov., and *Baoxiongia* gen. nov.) proposed in this study, MfGMB shared 37, 47, and 34 bacterial taxa at the species level with gut microbial biobanks of human^5^, ^6^, ^17^, ^18^, ^19^, mouse^20^, ^25^, and pig^21^, respectively. The MfGMB and other mammalian gut microbial biobanks^5^, ^6^, ^17^, ^18^, ^19^, ^20^, ^25^ provide diverse microbial resources and support causative and insightful studies on microbe–microbe and microbe–host interactions at the species level. The MfGMB reported previously unknown higher taxon, that is, *Zhongyuiaceae* fam. nov. within the order *Clostridiales*. *Zhongyuia ovalis* was the first isolates of the proposed family and its genome size was only 1.88 Mb. Smaller genomes are often associated with host parasitism^28^, ^29^. This genome of *Z. ovalis* encoded 1609 putative genes and 30.52% were functionally unknown genes. We observed that *Z. ovalis* assimilated N‐acetyl‐glucosamine, a component of mammalian soft bone and of some bacterial cell walls. *Z. ovalis* widely occurred in human GM (FO = 89.46%, RA = 0.0133%), yet, its functionality per se and its interactions with hosts remains to be investigated. We found that *Dysosmobacter* was a core genus (FO > 80% and RA > 0.1%) in *M. fascicularis* GMs. *Dysosmobacter welbionis* was first isolated from human faeces^30^ and so far the only species of genus *Dysosmobacter*. A recent study proved *D. welbionis* to be beneficial in the prevention of diet‐induced obesity^31^. The MfGMB reported two new species, *Dysosmobacter brevis* and *Dysosmobacter acutus*. The analysis of *D. brevis* genomes revealed the presence of butyrate synthesis pathways that occur in other bacteria^32^, ^33^, ^34^. Thus, *D. brevis* is a potential butyrate producer. Considering that butyrate serves as the main energy source for colonocytes^35^, butyrate‐producing bacteria play a key role in colonic health, including epithelial integrity; the new *Dysosmobacter* resources of MfGMB would support further causative studies and potential applications in probiotics.

## AUTHOR CONTRIBUTIONS

Conceived and designed the experiments: Shuang‐Jiang Liu. Performed the experiments: Danhua Li, Rexiding Abuduaini, Mengxuan Du, Yujing Wang. Analyzed the data: Chang Liu, Haizheng Zhu, Honghe Chen, Nan Zhou. Coordinated sample collections: Yong Lu, Qiang Sun. Conducted the microbial strain preservation: Yuhua Xin, Yuguang Zhou. Constructed the webpage and uploaded all the data: Linhuan Wu, Juncai Ma. Drafted the manuscript: Danhua Li, Chang Liu. Approved final version of manuscript: Chengying Jiang, Shuang‐Jiang Liu. All authors read and approved the final manuscript.

## CONFLICT OF INTERESTS

The authors declare no conflict of interests.

## ETHICS STATEMENT

The ethics application (ION‐2019043) was approved by the Institute of Neuroscience, Chinese Academy of Sciences.

## Supporting information

Supporting information.

Supporting information.

## Data Availability

The metadata generated and analyzed in this study are available as the following: all the polyphasic taxonomic information of 97 MfGMB species is available at MfGMB homepage (https://nmdc.cn/mfgmb/). All the 250 strains and their 16S rRNA gene sequences were accessible via MfGMB special page on CGMCC official website (https://www.cgmcc.net/english/mfgmb/). All the 16S rRNA gene  sequences and genomes generated in this study are accessible via GenBank using the accession numbers shown in Supplementary Datasets. The 16S rRNA gene amplicon data, metagenomic data, all the 16S rRNA gene sequences, and draft/complete genomes are accessible in NCBI via project PRJNA733006 (https://www.ncbi.nlm.nih.gov/bioproject/PRJNA733006) and NMDC via project NMDC10017790 (https://nmdc.cn/resource/genomics/project/detail/NMDC10017790).
