# Supplementary material for: The monkey microbial biobank brings previously uncultivated bioresources for nonhuman primate and human gut microbiomes
Source: mLife. 2022 May 24;1(2):210–7. doi: 10.1002/mlf2.12017 (PMC10989993; doi:10.1002/mlf2.12017)
Supplement: Supplementary file 2 — Supporting information. [file MLF2-1-210-s002.docx]

Supplementary Materials to:

**The Monkey microbial biobank brings previously uncultivated bioresources for non-human primate and human gut microbiomes**

Dan-Hua Li^#1^, Chang Liu^#1^, Rexiding Abuduaini^1,4^, Meng-Xuan Du^2^, Yu-Jing Wang^1,4^, Hai-Zhen Zhu^1^, Hong-He Chen^1^, Nan Zhou^1^, Yu-Hua Xin^1,6^, Lin-Huan Wu^1,5^, Jun-Cai Ma^1,5^, Yu-Guang Zhou^1,6^, Yong Lu^3^, Cheng-Ying Jiang^1,3^, Qiang Sun^3*^, Shuang-Jiang Liu^1,2,4*^

1. State Key Laboratory of Microbial Resources and Environmental Microbiology Research Center at Institute of Microbiology, Chinese Academy of Sciences, Beijing 100101, China

2. State Key Laboratory of Microbial Biotechnology, Shandong University, Qingdao 266237, China

3. Institute of Neuroscience, CAS Center for Excellence in Brain Science and Intelligence Technology, State Key Laboratory of Neuroscience, CAS Key Laboratory of Primate Neurobiology, Chinese Academy of Sciences, 200031, Shanghai, China

4. University of Chinese Academy of Sciences, Beijing 100049, China

5. *Microbial Resources and Big Data Center, Institute of Microbiology, Chinese Academy of Sciences, No.1 Beichenxi Road, Chaoyang District, Beijing 100101, P. R. China*

6. *China General Microorganism Culture Collection, Institute of Microbiology, Chinese Academy of Sciences, No.1 Beichenxi Road, Chaoyang District, Beijing 100101, P. R. China*

#Authors contributed equally.

*Corresponding authors:

Shuang-Jiang Liu, email to [liusj@im.ac.cn](mailto:liusj@im.ac.cn) or liusj@sdu.edu.cn

Qiang Sun, email to qsun@ion.ac.cn

**Materials and Methods**

**Sample collection for cultivation**

The use and care of cynomolgus monkeys (*M. fascicularis*) complied with the guideline of the Animal Advisory Committee at the Institute of Neuroscience, Chinese Academy of Sciences. The ethics application (ION-2019043) was approved by the Institute of Neuroscience, Chinese Academy of Sciences. The monkeys were fed captive diets consisted of monkey feed (wheat, corn, soybean meal, glucose, multiple vitamins, multiple minerals, amino acids, etc.); apples, vegetables, watermelon or other fruits; peanuts, melon seeds, raisins or other nuts. Total of 16 fresh stool samples from 7 captive cynomolgus macaques (*M. fascicularis*) at age of 0-5 years were collected for large-scale cultivation of gut microbes. In every case, fecal samples were collected immediately after defecation with a sterile collection tube and kept on dye ice during transportation to Institute of Microbiology, Chinese Academy of Sciences for further treatment.

**Sample treatment conditions**

The fresh feces samples were diluted with 5mL sterilized PBS solution and filtered through a 40 µm cell strainer (Falcon, USA) to prepare fecal suspensions for further usage. Then multiple sample treatment conditions were employed to detect higher bacterial diversity. A total of 73 treatment conditions were listed in Supplementary datasheet S1 and classified into six steps. Step1: the fecal suspensions were serially diluted with sterilized PBS solution and then directly inoculated on various media for anaerobically cultivation. Step 2: the fecal suspensions were treated by three different concentration of alcohol solution (35%, 50% and 70%) ^1^, then serially diluted and spread on various media for anaerobically cultivation. Step 3: the fecal suspensions were preincubated in anaerobic culture bottle for 1 or 3 days ^2^, then serially diluted and spread on various media for anaerobically cultivation. Step 4: the fecal suspensions were preincubated in anaerobic culture bottle for 3 days, then treated by 70% alcohol solution, serially diluted, and spread on various media for anaerobically cultivation. Step 5: the fecal suspensions were preincubated in anaerobic culture bottle for 3 days, then heated to 80 ^o^C for 25 min, serially diluted, and spread on various media for anaerobically cultivation. Step 6: the fecal suspensions were heated to 80 ^o^C for 25 min or 65 ^o^C for 25 min ^2,3^, then serially diluted, and spread on various media for anaerobically cultivation. These samples treatment conditions were applied to 16 stool samples and obtained 4,100 isolates as judged by 16S rRNA gene sequencing (Supplementary datasheet S3).

**Bacterial isolation, cultivation and storage strategy**

The pretreated fecal samples in the way described above were diluted serially. 100 μL of liquid from each of the dilution tubes were plated on the separate, labelled, and dry solid media. The recipes of 13 microbial media used in this study were listed in Supplementary datasheet S2. To increase the culturability of fastidious species, modified media were supplemented with reducing agent cysteine ^4^, redox dyes resazurin ^5^ and rumen fluid ^6^. The pH was adjusted and then the culture media were autoclaved. The sheep blood, carbohydrate mixture, vitamin K1 solution, Vitamin solution and Mineral solution were added to the autoclaved culture media stabilized at 60 ^o^C. The final culture media were poured to 99 mm petri dishes. The solutions formulations were also listed in Supplementary datasheet S2. The cultivation occurred at 37 ^o^C under strictly anaerobic conditions in an anaerobic chamber (Electrotek AW400SG workstation, West Yorkshire, UK) with N_2_ (85%), H_2_ (10%) and CO_2_ (5%) for 2-15 days. Well-separated single colonies arose on some of the agar plates. For isolating single colonies, a plate streaking method was applied. The single colonies were streaked with an inoculated loop into 12-well plates, which were incubated anaerobically at 37 ^o^C until colonies become visible. A simple and effective colony PCR procedure is employed ^7^. Cells from colonies were picked up from 12-well plates and resuspended into 5 μL NaOH/SDS lysis buffer (Amresco, USA). The lysates were next diluted to 100 µL distilled water and 5µL suspensions were used for PCR amplification of 16S rRNA gene with the universal primers 27F (5’-AGAGTTTGATCCTGGCTCAG-3’) and 1492R (5’- GGTTACCTTGTTACGACTT-3’). The colonies with single morphology and single 16S rRNA gene were expanded and inoculated in anaerobic tubes with 5 ml of liquid media. Repeatedly streaking the culture on agar plates until axenic culture obtained, the axenic culture was used for further characterization and preservation. 16S rRNA sequences similarities between isolates and the previously identified relatives (valid names only) were determined using EzBioCloud server ^8^. the 16S rRNA gene sequence similarity was used to identify and recognize a new species. The cutoff value is 98.7% ^9^. The isolates with 16S rRNA sequences similarities showing 98.7% or higher were selected as known species. The isolates (<98.7% 16S rRNA similarities) were labeled as potential novel species for further taxonomic purposes.

Considering the strains, while belonging to the same species, from different hosts or different environments always have genome diversity, the storage strategy was employed to ensure the strain-level diversity. Stool samples from 7 captive cynomolgus macaques (*M. fascicularis*) at different ages was used in this study. We deposited at least one representative strain of every identified taxon from different monkeys or different ages. No matter whether strains of these taxon had ever been preserved or not, strains were deposited to the China General Microbiological Culture Collection Center (CGMCC) or Korean Collection for Type Cultures (KCTC) for long-term preservation and public accessibility. The information of 250 strains deposited in CGMCC and the type/represented strains of the novel taxa were provided in Supplementary datasets and MfGMB special page on CGMCC (<http://www.cgmcc.net/english/mfgmb/>). For the storage, all the identified strains were cultured in liquid medium for 2 days. The 1 mL fresh culture was added to 1 mL PBS solutions with 65% (v/v) glycerol. Tubes were stored at -80 ^o^C for long-term preservation.

**Characterization of novel taxa**

A polyphasic taxonomic approach including phenotypic, biochemical, and physiological characteristics, phylogenetic analysis, phylogenomic tree and genome data was applied to characterize the novel taxa as described in our previous study ^10^. To be specific, cell morphology was determined by transmission electron microscopy (TEM) (JEM-1400; JEOL, Japan). The utilization of their sole carbon source was determined using the 96 well BIOLOG AN microplate (BIOLOG Inc., Hayward, CA, USA) which contained 95 different carbon substrates and one negative control ^11^. Complete 16S rRNA sequences of isolates were obtained using the universal primers above-mentioned. Multiple alignments of 16S rRNA gene sequences between isolated strains and the closely related organisms were performed using the CLUSTAL W ^12^. The phylogenetic trees were constructed by the neighbor-joining ^13^ according to Kimura’s two-parameter model ^14^ in MEGA version 6.0 ^15^ with 1000 bootstrap replications ^16^. The phylogenetic trees based on genome were also constructed using gtdb-tk with classify_wf command under default parameters ^17^. According to the phylogenomic tree, the closely related and available genomes were employed to calculate the average nucleotide identity (ANI) value using OAT software at http://www.ezbiocloud.net/sw/oat ^18^. The genomic distances, digital DNA-DNA hybridization (dDDH), were calculated by using the Genome-To-Genome Distance Calculator (GGDC; http://ggdc.dsmz.de/) ^19^. The percentage of conserved proteins (POCP) was calculated by the method previously described ^20^. Given the diversity of the isolated species, a strategy was necessary to identify the isolated novel species and meet the needs of microbial taxonomy. This strategy consisted of three main steps: 1) The proposed and generally accepted species boundary for 16S similarity, ANI and dDDH values are 98.7%, 95-96 and 70%, respectively ^9^. The strains formed a distinct clade in phylogenetic trees and with <98.7% 16S similarity, <95~96% ANI and <70% dDDH were identified as a new species. 2) The strains formed a distinct clade to the closely related species, with <94.5% 16S similarity or <50% POCP and with distinct phenotypic and biochemical characterization were identified as a new genus. 3) the strains formed a distinct clade to the closely related genus, with <90% 16S rRNA gene identity, and with distinct phenotypic and biochemical characterization were identified as a new family. The taxonomic information and genomes characterization of MfGMB were provided in Supplementary datasheet S5. The polyphasic taxonomic features of the novel taxa were described in Table 1 and reference ^21^.

**Genome sequencing and analysis**

A total of 97 genomes (93 draft genomes and 4 complete genomes) from 97 taxa including 32 new taxa and 65 known taxa were sequenced. For genome sequencing, genomic DNA was extracted using the Wizard Genomic DNA Purification Kit (Promega, USA). Agarose gels electrophoresis (1%) was used to monitor the DNA integrity. DNA concentration and purity were measured using Qubit 2.0 (Thermo Fisher Scientific, Waltham, USA) and Nanodrop One (Thermo Fisher Scientific, Waltham, USA) at the same time. Sequencing libraries were constructed using NEBNext Ultra DNA Library Prep Kit for Illumina (New England Biolabs, USA) following the protocol recommended. The library quality was assessed on the Qubit 3.0 Fluorometer (Life Technologies, Grand Island, NY) and Agilent 4200 (Agilent, Santa Clara, CA) system. For draft genome sequencing, the library was sequenced on an Illumina Hiseq X-ten platform and 150 bp paired-end reads were produced. The low-quality reads were filtered by the step of quality control. All good quality paired reads were assembled using the SPAdes software (v3.9.0) ^22^ into several contigs. The PacBio Sequel platform was used for sequencing complete genome sequencing. Single-Molecule Real-Time (SMRT) was performed on the Pacific Biosciences RSII sequencer (PacBio, Menlo Park, USA) according to standard protocols. Qualified genomic DNA was fragmented with G-tubes (Covaris) and end-repaired to prepare SMRTbell DNA template libraries (fragment size >10 Kb selected by bluepippin system) according to the manufacturer’s specification (PacBio, Menlo Park, USA). Library quality was detected by Qubit 3.0 Fluorometer (Life Technologies, Grand Island, NY) and Average fragment size was estimated on an Agilent 4200 (Agilent, Santa Clara, CA). The low-quality reads were filtered and the filtered reads were assembled by Unicycler ^23^ to generate one contig without gaps. The assembly results were optimized by Arrow software. Above-mentioned raw data of genomes were performed at Magigene (Guangzhou, China). Check M was used to assess the quality of microbial genomes recovered from isolates ^24^. Genomes with contamination>5% were further decontaminated using MAGpurify v2.1.2 ^25^. The prediction of coding genes were performed with glimmer3 ^26^ and Prodigal v2.6.3 ^27^, and the 16S rRNA genes were retrieved by RNAmmer v1.2 ^28^. The function annotations of all genomes were performed based on eggNOG database v5.0 by local emapper v1.0.3 (-m diamond) ^29^.

**Bacterial diversities of different host-derived culture collections**

We collected the taxonomic information of gut microbe cultures from different hosts as humans ^10,30-33^, mouse ^34,35^ Pig ^36^ for diversity comparison with MfGMB in this study. The 16S rRNA gene sequences were retrieved either from the publications ^10,34-36^ or from genome data ^28,30-33^ using RNAmmer v1.2. The genome-derived 16S rRNA gene sequences > 1.2 kb were retained for further analysis. The 16S rRNA gene sequences of novel taxa isolates/genomes from the same hosts were clustered using Usearch11 (command: -cluster_fast query.fasta -id 0.987 -centroids clustered.16S.fasta -uc clusters.uc) to reveal the nonredundant 16S rRNA gene sequences of species-level taxa in each host. We then analyzed the overlaps of species among hosts by calculation the Kimura 2-parameter model-based evolution distance between representative species-level 16S rRNA gene sequences from different hosts using MEGA version 6.0 ^15^. If the representative 16S rRNA gene sequences from different hosts had 16S rRNA gene sequence distance <0.013 to each other, they were regarded as the “shared” species, otherwise, the representativeness was defined as host-unique species. The unique and shared bacteria within MfGMB and the other investigated collections representing three different hosts were displayed using Venn and bar charts generated by Jvenn ^37^.

**Gut metagenome sequencing, collection and analysis**

The metagenomic DNA preparation and sequencing were performed by commercial company (MAGIGENE inc., China). Briefly, 5 of 16 fecal samples from captive cynomolgus macaques (*M. fascicularis*) used in cultivation were selected for metagenomes sequencing and the DNAs were extracted using QIAGEN DNA Stool Mini Kit (QIAGEN, Germany) following a standard protocol as recommended. Sequencing libraries were generated using NEBNext® Ultra™ DNA Library Prep Kit for Illumina (NEB, USA), and index codes were added to attribute sequences to each sample. The metagenomic DNAs were fragmented by sonication to a size of 350 bp, then the generated DNA fragments were end-polished, A-tailed, and ligated with the full-length adaptor for Illumina sequencing. After amplification, PCR products were purified (AMPure XP system) and libraries were analyzed for size distribution by Agilent2100 Bioanalyzer and quantified using real-time PCR. The clustering of the index-coded samples was performed on a cBot Cluster Generation System according to the manufacturer’s instructions. After cluster generation, the library preparations were sequenced on an Illumina HiSeq 2000 platform and paired-end reads were generated. Raw data was conducted using Readfq V8 ( https://github.com/cjfields/readfq）to acquire clean data. The clean Data were blast to the host database using Bowtie2.2.4 software64 to filter the reads that are of host origin. The 20 publically-available metagenomes of fecal samples from cynomolgus macaques used for the construction of *M. fascicularis* gut microbiome gene catalog ^38^ were achieved (data accessions are available in “Data Availability”) and analyzed together with the datasets sequenced in this study. The 1,129 publicly available metagenomic data samples of health human GMs were selected and processed as described in previous work ^10^ The accessions of the 1,129 samples are listed in Supplementary datasets. The distribution of novel taxa among metagenomes were estimated by Kraken 2 v2.0.9-beta ^39^. A customized Kraken 2 database was constructed for taxonomic annotation by supplementation of 37 novel taxon genomes from MfGMB into the previous-constructed database GTDB-species_vhGMB ^10^ to generate a new customized database, the GTDB_species_vMfGMB, which were further used for taxonomical annotation of metagenomes. Default parameters were used for each software unless otherwise specified.

**The 16S rRNA gene amplicon sequencing and analysis**

To understand the gut microbiota compositions of experimental *M. fascicularis*, 161 fecal samples from above-mentioned 7 captive cynomolgus macaques (*M. fascicularis*) aged 0-5 years were collected and their metagenomic DNAs were isolated using QIAGEN DNA Stool Kit (QIAGEN, Germany) following standard protocol as recommended. With the primers F341 (5’-CCTACGGGRSGCAGCAG-3’) and R806 (5’-GGACTACVVGGGTATCTAATC-3’) containing the barcode, the V3-V4 regions of 16S rRNA gene was ampliated by SequalPrep™ Long PCR Kit following standard protocol. Amplicons after 30-cycle PCR amplification were then used for the generation of sequencing libraries using NEBNext Ultra II DNA Library Prep Kit for Illumina (New England Biolabs, MA, USA). The library was sequenced on an Illumina Miseq platform. All clean data was further processed using the 64-bit Usearch software61 v11 in accordance with the recommended uparse-based pipeline (<https://drive5.com/usearch/manual/uparse_pipeline.html>). The only modification of the procedure was that an additional chimera removal step was introduced after OTU sequences were generated with the command “-uchime2_ref” against SILVA v132 database. The ZOTUs (zero-radius operational taxonomic units) were achieved by denoising the unique sequences by UNOISE algorithm. The ZOTUs were annotated with the customized LTP (Living tree program) database LTP_vbiobank. The LTP_vbiobank database was generated by supplementing the 16S rRNA gene sequences of new taxa in hGMB ^10^, mGMB ^35^ and MfGMB into LTP version 132. An ZOTU table was constructed to include the information of abundance and annotation for each ZOTU after normalization to the minimum library size of sample (Supplementary datasheet S8). The alpha and beta diversity for the amplicon dataset was analyzed and visualized using MicrobiomeAnalyst ^40^. To define the dominant, common and core genera for *M. fascicularis* gut microbiota, the relative abundance (RA) and frequency of occurrence (FO) of each genus was calculated as described in our previous publication ^10^. The average RA> 0.1% was the criterion to define dominant genera, while the average FOs>80% was the criteria for definition of common genera. The genus with RA>0.1% and FO>80% was defined as core genus. The distribution of dominant taxa in global human GMs were displayed as box-and-whiskers plots while the common taxa were displayed as bar charts.

**Gene catalog construction and analysis**

The *M. fascicularis* global gut microbial gene catalog containing 1,991,169 nonredundant genes ^38^ were downloaded and reannotated with eggNOG database v5.0 by emapper v1.0.3 (-m diamond) ^29^ and generated indexed databases for each gene catalogs with DIAMOND v0.9.24 (makedb command) ^41^. The nonredundant gene catalog MfGMB.catalog was generated from 97 MfGMB genomes using CD-HIT software v4.5.8 ^42^ (-o out.file -c 0.95 -aS 0.9 -n 5 -M 64000 -T 48). The MfGMB.catalog containing 313,603 nonredundant genes were then annotated with eggNOG database v5.0 by emapper v1.0.3 ^29^. The eggNOG orthologs and KOs were summarized from the eggNOG annotation results. The BLAST analysis of single genomes and MfGMB.catalog against global gene catalog were performed using DIAMOND blastp with a sequence identity cut-off value of 40% and query coverage cut-off value of 70%. (--query-cover 70 -id 40 --more-sensitive -f 6 qseqid sseqid pident length qlen slen qcovhsp evalue qseq full_sseq mismatch gapopen qstart qend sstart send). The KO-based and BLAST_based coverages of genes were tabularized and displayed as described in previous publication.

**Statistical analysis**

All statistical analyses were performed using IBM SPSS Statistics 20. All the box-and–whiskers plots, bar charts and accumulative curves were generated using Graphpad Prism v6 ^43^ unless indicated otherwise. Comparison of two groups of data was statistically assessed with Mann–Whitney U test, while comparison of multi groups (>2) of data was evaluated by Kruskal-Willis test. *P* < 0.05 was considered being statistically significant (*p* < 0.05: *, *p* < 0.01: **, *p* < 0.001: ***). All the calculations as RA, FO and coverage were expressed in the form of mean ± SD unless indicated otherwise. The boxplots showed the median values and whiskers extending to include all the valid data denoted by Turkey test.

**References**

1. Afouda P, Hocquart M, Pham TP, Kuete E, Ngom, II, Dione N*, et al.* Alcohol pretreatment of stools effect on culturomics. Sci Rep. 2020;10(1):5190.

2. Lagier JC, Hugon P, Khelaifia S, Fournier PE, La Scola B, Raoult D. The rebirth of culture in microbiology through the example of culturomics to study human gut microbiota. Clin Microbiol Rev. 2015;28(1):237-64.

3. Mailhe M, Ricaboni D, Vitton V, Gonzalez JM, Bachar D, Dubourg G*, et al.* Repertoire of the gut microbiota from stomach to colon using culturomics and next-generation sequencing. BMC Microbiol. 2018;18(1):157.

4. Gaspar AJ, Faber JE, Jr. A transparent solid medium for growth enhancement of Pasteurella tularensis. Appl Microbiol. 1962;10(1):90-2.

5. Mauerhofer LM, Pappenreiter P, Paulik C, Seifert AH, Bernacchi S, Rittmann SKR. Methods for quantification of growth and productivity in anaerobic microbiology and biotechnology. Folia Microbiol (Praha). 2019;64(3):321-60.

6. Lagier JC, Khelaifia S, Alou MT, Ndongo S, Dione N, Hugon P*, et al.* Culture of previously uncultured members of the human gut microbiota by culturomics. Nat Microbiol. 2016;1:16203.

7. Packeiser H, Lim C, Balagurunathan B, Wu J, Zhao H. An extremely simple and effective colony PCR procedure for bacteria, yeasts, and microalgae. Appl Biochem Biotechnol. 2013;169(2):695-700.

8. Yoon SH, Ha SM, Kwon S, Lim J, Kim Y, Seo H*, et al.* Introducing EzBioCloud: a taxonomically united database of 16S rRNA gene sequences and whole-genome assemblies. Int J Syst Evol Microbiol. 2017;67(5):1613-17.

9. Chun J, Oren A, Ventosa A, Christensen H, Arahal DR, da Costa MS*, et al.* Proposed minimal standards for the use of genome data for the taxonomy of prokaryotes. Int J Syst Evol Microbiol. 2018;68(1):461-66.

10. Liu C, Du MX, Abuduaini R, Yu HY, Li DH, Wang YJ*, et al.* Enlightening the taxonomy darkness of human gut microbiomes with a cultured biobank. Microbiome. 2021;9(1):119.

11. Preston-Mafham J, Boddy L, Randerson PF. Analysis of microbial community functional diversity using sole-carbon-source utilisation profiles - a critique. FEMS Microbiol Ecol. 2002;42(1):1-14.

12. Thompson JD, Higgins DG, Gibson TJ. CLUSTAL W: improving the sensitivity of progressive multiple sequence alignment through sequence weighting, position-specific gap penalties and weight matrix choice. Nucleic Acids Res. 1994;22(22):4673-80.

13. Saitou N, Nei M. The neighbor-joining method: a new method for reconstructing phylogenetic trees. Mol Biol Evol. 1987;4(4):406-25.

14. Kimura M. A simple method for estimating evolutionary rates of base substitutions through comparative studies of nucleotide sequences. J Mol Evol. 1980;16(2):111-20.

15. Tamura K, Stecher G, Peterson D, Filipski A, Kumar S. MEGA6: Molecular Evolutionary Genetics Analysis version 6.0. Mol Biol Evol. 2013;30(12):2725-29.

16. Felsenstein J. CONFIDENCE LIMITS ON PHYLOGENIES: AN APPROACH USING THE BOOTSTRAP. Evolution. 1985;39(4):783-91.

17. Chaumeil PA, Mussig AJ, Hugenholtz P, Parks DH. GTDB-Tk: a toolkit to classify genomes with the Genome Taxonomy Database. Bioinformatics. 2020;36(6):1925-27.

18. Lee I, Ouk Kim Y, Park SC, Chun J. OrthoANI: An improved algorithm and software for calculating average nucleotide identity. Int J Syst Evol Microbiol. 2016;66(2):1100-03.

19. Meier-Kolthoff JP, Auch AF, Klenk HP, Göker M. Genome sequence-based species delimitation with confidence intervals and improved distance functions. BMC Bioinformatics. 2013;14:60.

20. Qin QL, Xie BB, Zhang XY, Chen XL, Zhou BC, Zhou J*, et al.* A proposed genus boundary for the prokaryotes based on genomic insights. J Bacteriol. 2014;196(12):2210-15.

21. Li DH, Abuduaini R, Du MX, Wang YJ, Chen HH, Zhou N, *et al.* *Alkaliphilus flagellatus* sp. nov., *Butyricicoccus intestinisimiae* sp. nov., *Clostridium mobile* sp. nov., *Clostridium simiarum* sp. nov., *Dysosmobacter acutus* sp. nov., *Paenibacillus brevis* sp. nov., *Peptoniphilus ovalis* sp. nov., and *Tissierella simiarum* sp. nov., isolated from monkey feces. Int J Syst Evol Microbiol. 2021.

22. Bankevich A, Nurk S, Antipov D, Gurevich AA, Dvorkin M, Kulikov AS*, et al.* SPAdes: a new genome assembly algorithm and its applications to single-cell sequencing. J Comput Biol. 2012;19(5):455-77.

23. Wick RR, Judd LM, Gorrie CL, Holt KE. Unicycler: Resolving bacterial genome assemblies from short and long sequencing reads. PLoS Comput Biol. 2017;13(6):e1005595.

24. Parks DH, Imelfort M, Skennerton CT, Hugenholtz P, Tyson GW. CheckM: assessing the quality of microbial genomes recovered from isolates, single cells, and metagenomes. Genome Res. 2015;25(7):1043-55.

25. Nayfach S, Shi ZJ, Seshadri R, Pollard KS, Kyrpides NC. New insights from uncultivated genomes of the global human gut microbiome. Nature. 2019;568(7753):505-10.

26. Delcher AL, Bratke KA, Powers EC, Salzberg SL. Identifying bacterial genes and endosymbiont DNA with Glimmer. Bioinformatics. 2007;23(6):673-79.

27. Hyatt D, Chen GL, LoCascio PF, Land ML, Larimer FW, Hauser LJ. Prodigal: prokaryotic gene recognition and translation initiation site identification. BMC Bioinformatics. 2010;11.

28. Lagesen K, Hallin P, Rødland EA, Staerfeldt HH, Rognes T, Ussery DW. RNAmmer: consistent and rapid annotation of ribosomal RNA genes. Nucleic Acids Res. 2007;35(9):3100-8.

29. Huerta-Cepas J, Szklarczyk D, Forslund K, Cook H, Heller D, Walter MC*, et al.* eggNOG 4.5: a hierarchical orthology framework with improved functional annotations for eukaryotic, prokaryotic and viral sequences. Nucleic Acids Research. 2016;44(D1):D286-D93.

30. Forster SC, Kumar N, Anonye BO, Almeida A, Viciani E, Stares MD*, et al.* A human gut bacterial genome and culture collection for improved metagenomic analyses. Nat Biotechnol. 2019;37(2):186-92.

31. Zou Y, Xue W, Luo G, Deng Z, Qin P, Guo R*, et al.* 1,520 reference genomes from cultivated human gut bacteria enable functional microbiome analyses. Nat Biotechnol. 2019;37(2):179-85.

32. Poyet M, Groussin M, Gibbons SM, Avila-Pacheco J, Jiang X, Kearney SM*, et al.* A library of human gut bacterial isolates paired with longitudinal multiomics data enables mechanistic microbiome research. Nat Med. 2019;25(9):1442-52.

33. Browne HP, Forster SC, Anonye BO, Kumar N, Neville BA, Stares MD*, et al.* Culturing of 'unculturable' human microbiota reveals novel taxa and extensive sporulation. Nature. 2016;533(7604):543-46.

34. Lagkouvardos I, Pukall R, Abt B, Foesel BU, Meier-Kolthoff JP, Kumar N*, et al.* Corrigendum: The Mouse Intestinal Bacterial Collection (miBC) provides host-specific insight into cultured diversity and functional potential of the gut microbiota. Nat Microbiol. 2016;1(11):16219.

35. Liu C, Zhou N, Du MX, Sun YT, Wang K, Wang YJ*, et al.* The Mouse Gut Microbial Biobank expands the coverage of cultured bacteria. Nat Commun. 2020;11(1):79.

36. Wylensek D, Hitch TCA, Riedel T, Afrizal A, Kumar N, Wortmann E*, et al.* A collection of bacterial isolates from the pig intestine reveals functional and taxonomic diversity. Nat Commun. 2020;11(1):6389.

37. Bardou P, Mariette J, Escudie F, Djemiel C, Klopp C. jvenn: an interactive Venn diagram viewer. BMC Bioinformatics. 2014;15.

38. Li X, Liang S, Xia Z, Qu J, Liu H, Liu C*, et al.* Establishment of a *Macaca fascicularis* gut microbiome gene catalog and comparison with the human, pig, and mouse gut microbiomes. Gigascience. 2018;7(9).

39. Wood DE, Lu J, Langmead B. Improved metagenomic analysis with Kraken 2. Genome Biology. 2019;20(1).

40. Chong J, Liu P, Zhou G, Xia J. Using MicrobiomeAnalyst for comprehensive statistical, functional, and meta-analysis of microbiome data. Nat Protoc. 2020;15(3):799-821.

41. Buchfink B, Xie C, Huson DH. Fast and sensitive protein alignment using DIAMOND. Nature Methods. 2015;12(1):59-60.

42. Fu LM, Niu BF, Zhu ZW, Wu ST, Li WZ. CD-HIT: accelerated for clustering the next-generation sequencing data. Bioinformatics. 2012;28(23):3150-52.

43. Basham B. Graphpad Prism. Biotechnology Software & Internet Journal. 1997;14(6):14-17.
